# Lazarus Phenomenon or the Return from the Afterlife—What We Know about Auto Resuscitation

**DOI:** 10.3390/jcm12144704

**Published:** 2023-07-15

**Authors:** Piotr Rzeźniczek, Agnieszka Danuta Gaczkowska, Anna Kluzik, Marcin Cybulski, Alicja Bartkowska-Śniatkowska, Małgorzata Grześkowiak

**Affiliations:** 1 Department of Teaching Anesthesiology and Intensive Therapy, Poznan University of Medical Sciences, 61-861 Poznan, Poland; 2 Department of Anesthesiology, Intensive Therapy and Pain Treatment, Poznan University of Medical Sciences, 60-806 Poznan, Poland; 3 Department of Clinical Psychology, Poznan University of Medical Sciences, 60-806 Poznan, Poland; 4 Department of Pediatric Anesthesiology and Intensive Therapy, Poznan University of Medical Sciences, 60-806 Poznan, Poland

**Keywords:** Lazarus phenomenon, Lazarus syndrom, autoresuscitation

## Abstract

Autoresuscitation is a phenomenon of the heart during which it can resume its spontaneous activity and generate circulation. It was described for the first time by K. Linko in 1982 as a recovery after discontinued cardiopulmonary resuscitation (CPR). J.G. Bray named the recovery from death the Lazarus phenomenon in 1993. It is based on a biblical story of Jesus’ resurrection of Lazarus four days after confirmation of his death. Up to the end of 2022, 76 cases (coming from 27 countries) of spontaneous recovery after death were reported; among them, 10 occurred in children. The youngest patient was 9 months old, and the oldest was 97 years old. The longest resuscitation lasted 90 min, but the shortest was 6 min. Cardiac arrest occurred in and out of the hospital. The majority of the patients suffered from many diseases. In most cases of the Lazarus phenomenon, the observed rhythms at cardiac arrest were non-shockable (Asystole, PEA). Survival time after death ranged from minutes to hours, days, and even months. Six patients with the Lazarus phenomenon reached full recovery without neurological impairment. Some of the causes leading to autoresuscitation presented here are hyperventilation and alkalosis, auto-PEEP, delayed drug action, hypothermia, intoxication, metabolic disorders (hyperkalemia), and unobserved minimal vital signs. To avoid Lazarus Syndrome, it is recommended that the patient be monitored for 10 min after discontinuing CPR. Knowledge about this phenomenon should be disseminated in the medical community in order to improve the reporting of such cases. The probability of autoresuscitation among older people is possible.

## 1. The Death and Resurrection of Lazarus

Now a certain man was sick, Lazarus of Bethany, the village of Mary and her sister Martha. It was the Mary who anointed the Lord with ointment, and wiped His feet with her hair, whose brother Lazarus was sick. So the sisters sent word to Him, saying, “Lord, behold, he whom You love is sick”. But when Jesus heard this, He said, “This sickness is not to end in death, but for the glory of God…”. Now Jesus had spoken of his death, but they thought that He was speaking of literal sleep. So Jesus then said to them plainly, “Lazarus is dead, and I am glad for your sakes that I was not there, so that you may believe; but let us go to him”. So when Jesus came, He found that he had already been in the tomb four days. So Jesus, again being deeply moved within, came to the tomb. Now it was a cave, and a stone was lying against it. Jesus said, “Remove the stone”. Martha, the sister of the deceased, said to Him, “Lord, by this time there will be a stench, for he has been dead four days”. Jesus said to her, “Did I not say to you that if you believe, you will see the glory of God?”. So they removed the stone. Then Jesus raised His eyes, and said, “Father, I thank You that You have heard Me. “Lazarus, come forth”. The man who had died came forth, bound hand and foot with wrappings, and his face was wrapped around with a cloth. Jesus said to them, “Unbind him, and let him go”. John 11: New American Standard Bible 1995 (NASB95) [1].

## 2. Introduction

The phenomenon of Lazarus syndrome presented in this article, otherwise known as autoresuscitation, is a phenomenon known in the literature, although no sufficient scientific evidence pointing to the specific cause of such phenomenon has been demonstrated so far. The intention of the authors is to present to the medical community a problem connected to the spontaneous return of life functions after the patient’s death.

Throughout centuries there has always been a fear among people that one may wake up in the coffin after being buried. That concern stemmed from the difficulty of confirming death.

There are known cases in the literature describing patients who, after being pronounced dead, somehow became alive again. This phenomenon resulted in the past from a lack of possibility to exclude the so-called reversible causes of cardiac arrest, among others, such as hypothermia or poisoning included in the updated guidelines of the European Resuscitation Council (ERC) 2021 [2]. In the past special coffins were even constructed, which enabled the communication of the buried deceased people with those alive should the signs of life return to them.

Nowadays, the declaration of death is not as difficult as before. Currently, the technical improvements enable us to perform thorough diagnostics, which limit the possibility of an “error” and of coming back to life of a patient who had been pronounced dead.

## 3. Lazarus Phenomenon—What Do We Know?

The cases of patients in which life functions, including heart rate, have been restored after death have been presented in world literature. Initially, this phenomenon became known as “autoresuscitation” and was described for the first time in 1982 by K. Linko et al. [3]. The definition of this phenomenon as “Lazarus Syndrome/Phenomenon” was proposed by J.G Bray in 1993, influenced by the Bible story about Lazarus, who was resurrected by Jezus 4 days after his death [4]. In his 2004 article, E.F. Wijdicks specified that autoresuscitation is a phenomenon of the heart, which can resume spontaneous activity and thus generate a heartbeat [5]. It has been noted that “autoresuscitation” or “coming back to life” can occur following insufficient resuscitation or lack thereof when the circulation is restored by itself (spontaneously).

The data from the literature point to various times of returning of the life functions, from a few seconds to several dozen minutes. A case of autoresuscitation after 30 s was reported by JP Duff [6]; however, the longest recorded time of resuming all life function presented in literature so far—180 min—has been described by AT Guyen [7]. The greatest number of autoresuscitation cases described as Lazarus Syndrome took place within a few minutes time frame and this is why most authors recommend at least 10 min monitoring of the patient’s ECG recording following the cessation of all resuscitation procedures in order to exclude autoresuscitation.

The following three electronic databases were searched, containing all reports published from the year 1982 until 31 December 2022: PubMed, Scopus, and Web of Science. The phrases used for analyses were: “Lazarus phenomenon” or “Lazarus or phenomenon”, “Lazarus syndrome or Lazarus or syndrome”, and “Autoresuscitation or Auto-resuscitation”. Each database was independently reviewed by two members of the research team, and the results were compared. Duplicated records were excluded, as well as review articles. In the remaining original reports we found, we searched case reports of Lazarus syndrome. Finally, we found 10,627 records, 1650 entries met the criteria for inclusion in the initial analysis, and after further exclusion of 1494 reports, we received 65 publications in which 76 cases of Lazarus syndrome were described.

**Figure 1:**
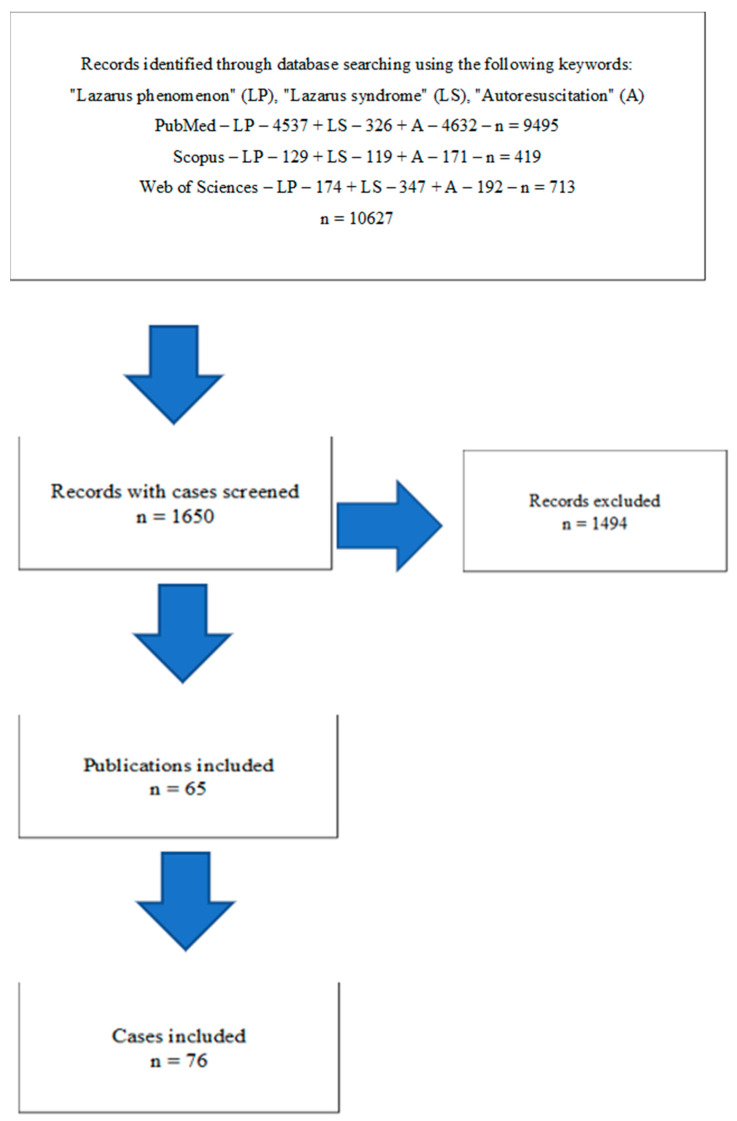
Flowchart of case selection.

Figure 1 illustrates how the data was collected.

## 4. Presumed Causes of Lazarus Syndrome

In this review article, we want to present the current state of knowledge about Lazarus Syndrome.

At this point, it is not known precisely what factors lead to autoresuscitation. Based on available publications, the factors that can be taken into account include hyperventilation, alkalosis, auto-PEEP (positive end-expiratory pressure), delayed drug action, hyperkalemia, unnoticeable vital signs as well as metabolic disorders [8,9].

**Hyperventilation** means the state of over-ventilation of a patient resulting from the administration of too much of the tidal volume with excessive frequency. It leads to a decrease in partial pressure of carbon dioxide (pCO_2_) and further to the development of respiratory alkalosis. It results in a shift in the hemoglobin dissociation curve to the left and reduced oxygen delivery to the tissues. The consequence of hyperventilation is cerebral vasoconstriction, which can lead to hypoxia of the central nervous system. It seems that the most important mechanism in Lazarus syndrome, which should be considered during hyperventilation, is a shortening of the exhalation time, leading to increased intrathoracic pressure and decreased venous return with a subsequent decrease in cardiac output. Decreased venous return translates directly to a slower delivery of drugs to the central circulation (and thus to delayed drug action, which is cited as another mechanism). When death is confirmed, resuscitation is discontinued, and then the patient’s “overventilation” state may be reversed. Generated due to excessive hyperventilation, the pressure in the chest is lowered, and perhaps by this mechanism, the heart can restart its activity. Cases of hyperventilation as a mechanism underlying Lazarus syndrome have been reported in children [6].

**Auto-PEEP** (Positive End Expiratory Pressure) [10], i.e., positive end-expiratory pressure in the airway, a type of air trap, which is predisposed by factors such as shortening the expiration time by increasing the respiratory rate, tidal volume, or inspiratory time, which is characteristic of hyperventilation and, similarly to hyperventilation, may lead to an increase in intrathoracic pressure, which may result in impaired venous return to the heart and a decrease in its output [11,12]. In this case, venous return is also reduced and may also slow down the distribution of drugs to the central circulation, referred to as the delayed-action mechanism. The confirmation of the above causes—i.e., hyperventilation and auto-PEEP—may be the case of the so-called incomplete Lazarus syndrome in a 7-day-old newborn admitted to the pediatric intensive care unit after aortic coarctation surgery [11]. Eight hours after surgery, the newborn in the ward had a significant drop in blood pressure (MAP 20–25 mmHg), with a heart rate of 70–80/min. Ventilation with a self-inflating bag was introduced, but the hypotension deepened, and only after the cessation of mechanical ventilation did the arterial pressure begin to increase. Suspecting the negative impact of excessive ventilation on the circulatory system, the number of breaths from the ventilator and PEEP were reduced, and within a few seconds, the return of a well-palpable pulse and normal sinus rhythm was observed. Cessation of mechanical ventilation and subsequent decrease in respiratory parameters led to the normalization of arterial pressure. This confirms one of the presumed causes of autoresuscitation—that is, hyperventilation, dynamic hyperinflation of the lungs together with air trapping (which in the described newborn was also confirmed in a chest radiograph). The following are also cited as the causes of the Lazarus phenomenon: lack of observation of vital signs (in the case of, for example, hypothermia), poisoning, and hyperkalemia. However, they are all listed in the European Resuscitation Guidelines (ERC 2021) as reversible causes of cardiac arrest that should be considered during resuscitation. If confirmed, the patient must be treated appropriately and cannot be pronounced dead.

Based on current data from the literature, we have described above the most likely causes of Lazarus syndrome, which have not been proven so far. We included the others as “controversial” because they are part of the reversible causes of cardiac arrest (which, according to the ERC 2021 guidelines starting from letter “H”, are: hypoxia, hypo-/hyperthermia, hypovolemia, hypo-/hyperkalemia, and metabolic disturbances [2]) and cannot be considered as separate causes.

Based on the available literature on the subject, some of the “controversial” causes of the Lazarus phenomenon will be discussed below.

A patient who shows no minimal vital signs, where a probable cause may be even hypothermia [13], presents with a controversial situation because hypothermia is a reversible cause of cardiac arrest, and only its exclusion or an increase in the patient’s body temperature may justify the declaration of death. Nevertheless, there are scientific reports which describe it as one of the reasons for the Lazarus phenomenon. In the case of hypothermia, biochemical processes are slowed down, which translates into the slowdown of basic life functions—virtually imperceptible breathing and pulse. Hypothermia is a state of decreased core body temperature with the distinction of several stages: mild, moderate, and severe hypothermia. In severe hypothermia (below 28 °C), all life processes slow down significantly with bradycardia, dilated pupils, lack of pupil reaction to light, and cardiac arrhythmias leading to cardiac arrest [14]. The above symptoms of hypothermia (especially in the deep stage) are identical to the clinical symptoms occurring in clinical death. Clinical death is a reversible condition. Resuscitation procedures in hypothermia, their length, drug distribution, etc., differ significantly from standard procedures [2]. In this case, it is important to consider the circumstances in which cardiac arrest occurred, the quality and conditions of resuscitation, the experience of medical personnel in recognizing minimal signs of life, proper patient monitoring, and consideration of advanced diagnostic techniques in making a decision to continue or withdraw from resuscitation.

Other causes of the Lazarus phenomenon are poisoning, i.e., a broadly understood phenomenon associated with the presence of harmful substances, poisons, drugs, etc., in the body. Toxic substances interact with compounds involved in physiological processes, disrupting their proper functioning at the systemic, cellular, or subcellular (mitochondrial) level. Diagnosis of poisoning is not always straightforward, and the supply of antidotes in such situations is often difficult and, in some situations, impossible [15]. Like hypothermia, poisoning is one of the reversible causes of cardiac arrest.

Hyperkalemia was mentioned in the cited literature as one of the causes of cardiac arrest in the course of renal failure and was also considered among the causes of the Lazarus phenomenon. Excessive levels of potassium ions lead to myocardial dysfunction in the sodium-potassium pump mechanism, which results in life-threatening arrhythmias, including cardiac arrest [16].

In the interpretation of the causes of autoresuscitation, the fact of the coexistence of many diseases in the patient is emphasized (e.g., cancer, cardiovascular diseases—e.g., cardiomyopathy [17], ischemic heart disease, sepsis [18]), as well as the patient’s advanced age [19]—the older the patient, the worse the prognosis, which undoubtedly affects survival after resuscitation.

## 5. Cases of Lazarus Phenomenon Documented in Literature

The first systematic study of cases of autoresuscitation from the world literature appeared in 2010 when K. Hornby presented 32 documented case reports of Lazarus syndrome [20]. Following him, in the same year, a group of German authors collected and expanded the set of described cases by another—a total of 42 [21]. In 2018, L. Hornby updated the list of global cases of autoresuscitation [22], and H. Herff described patients who developed Lazarus syndrome in German-speaking countries [23]. In 2020, L. Gordon et al., in a review article, presented a study of 65 cases of the Lazarus phenomenon from the years 1982 to 2018 based on medical databases: Google Scholar, Medline, and PubMed [24]. In 2021, M. Grzeskowiak et al. collected all 66 documented cases of the Lazarus syndrome up till the end of 2018 from 24 countries using medical databases: PubMed, Clinical Key, Scopus, and Web of Science and conducted a statistical analysis based on all available data on patients. They showed correlations between the duration of resuscitation and patient survival—the Lazarus phenomenon was more frequent in those patients who were resuscitated longer [25].

The problem of autoresuscitation is mainly dealt with by medical professionals, and therefore it is very important to further explore the causes of autoresuscitation. For the purposes of the previous article [25], we conducted a meta-analysis of medical databases until the end of 2018. We are currently continuing our research until the end of 2022 in order to present further cases of patients with the Lazarus phenomenon.

From the beginning of 2019 until the end of 2022, there were further reports, and 11 new cases of autoresuscitation were described. The phenomenon of Lazarus has been described in patients of both sexes, of all ages, in both adults and children. To sum up—by the end of 2022, seventy-six cases of the Lazarus phenomenon have been documented in the literature—in thirty-nine men, thirty-one women, and in six patients, the gender was not specified—in sixty-eight adults and eight children.

This is irrefutable proof that the Lazarus phenomenon occurs every year in the world, and more cases are being reported, but the problem, in our opinion, is the lack of education of medical staff on this issue and the undetermined management of such a patient (lack of guidelines).

The oldest two patients were 97 years old [26,27], and the youngest was 9 months old [6]. The longest resuscitation time was 90 min [28], and the shortest was 6 min [29]. Reports come from 27 countries. Based on the collected data from the literature on the Lazarus phenomenon, the rhythms that occurred during resuscitation were mostly non-shockable rhythms [30,31,32,33]. Asystole was the predominant mechanism of cardiac arrest and occurred in as many as 43 patients, of which, in 41, it was the only rhythm during the entire resuscitation. Pulseless electrical activity (PEA) was the only rhythm that occurred in 22 patients. Asystole associated with a different rhythm occurred in two patients: a 97-year-old man (with ventricular fibrillation) [26] and a 62-year-old man (with PEA) [34]. It would seem that these “unfavorable, poor prognosis” non-shockable rhythms will not lead to the return of vital functions. Meanwhile, the defibrillation rhythm described in patients with Lazarus syndrome was only ventricular fibrillation (VF), which appeared in two patients (and in one of them, a 66-year-old man [35], it persisted throughout the duration of rescue activities, i.e., 30 min. Statistical analysis conducted by M. Grześkowiak et al. showed no relationship taking into account gender (11.43% vs. 17.39%; *p* = 0.70) and heart rate (33.33% vs. 14.29%; *p* = 0.17) of patients who experienced the phenomenon. In most of them, cardiac arrest was not of cardiac origin (n = 18/30) [25].

The survival time of patients after autoresuscitation described in the literature (understood as the period in which the patients had their own vital functions preserved and did not require connection to medical equipment) ranged from a few minutes to full recovery. Based on the available literature, it is known that 25 out of 71 patients regained consciousness (however, it is not precisely specified, some patients opened their eyes, and others made sounds) [25].

## 6. Recovery from Autoresuscitation

Full recovery, without neurological deficits, after an episode of autoresuscitation was reported in six patients. Among the documented cases of recovery was a 36-year-old woman who had a PEA-induced cardiac arrest. Resuscitation procedures were performed for 25 min before she was pronounced dead [36]. Spontaneous return of circulation occurred 3 min after the end of rescue operations. Six months after the episode of resuscitation, the woman returned to her regular activities at home.

Another patient who was diagnosed with Lazarus syndrome and who recovered was a 66-year-old man treated for hypertension and type 2 diabetes. The patient reported severe chest pain immediately before cardiac arrest, during which ventricular fibrillation was detected. Resuscitation procedures were implemented during which defibrillation was performed. During transport to the hospital, there was a temporary return of circulation. Resuscitation was continued in the hospital due to defibrillation-resistant ventricular fibrillation, but after 45 min, a decision was made to stop resuscitation. Next, the decision was made to turn off the ECG monitor. However, PEA rhythm and the presence of agonal respirations were noted. After 5 min, the respiratory rate increased, and a pulse was palpable. Twelve-lead ECG confirmed inferior wall infarction. The percutaneous coronary intervention was performed in the reference center, after which the patient was transferred to the intensive care unit, where he was extubated the next day. On the ninth day, the patient without neurological deficits was discharged home. Later, he also returned to normal physical activity [37].

Another example of recovery after an episode of autoresuscitation was an 84-year-old man operated on for an abdominal aortic aneurysm after previous bypass implantation. The man suffered from mild senile dementia. Cardiac arrest occurred in out-of-hospital conditions. Rescue operations were not carried out until the arrival of the emergency medical team (EMS), which upon arrival, confirmed cardiac arrest in the VF mechanism. During 15 min of resuscitation, defibrillation was performed four times. Between the second and third shock, the rhythm temporarily changed to PEA, then returned to VF. Return of spontaneous circulation and breathing occurred 5 min after the end of CPR. The unconscious patient was brought to a medical facility where diagnostics were performed. The tests showed a left branch block in the hospital. The patient, who was circulatory and respiratory efficient with preserved cognitive functions, was discharged home. According to the authors of the report, a man who survived Lazarus syndrome was in good general condition with slight cognitive impairments one year after the event [38].

## 7. Lazarus Phenomenon in Children

Spontaneous return of circulation after rescue operations in children is extremely rare. As of the end of 2022, only eight cases of self-resuscitation have been documented in this age group. The Lazarus Syndrome was described in an 18-month-old boy born prematurely at 24 weeks of gestation [39] who, for unknown reasons, suffered a cardiac arrest due to asystole. The rhythm during resuscitation changed to PEA. Resuscitation activities were carried out, during which five doses of adrenaline were administered intravenously. After this time, rescue operations were discontinued, and 6 min later, the circulation and consciousness returned spontaneously. This is the longest time recorded in children. The patient was then transferred to the pediatric intensive care unit, where he was extubated after four days. He died a year later with serious neurological disorders.

Another patient with Lazarus syndrome was a 10-year-old girl with mental retardation, cerebral palsy, microcephaly, quadriplegic spasticity, and central obstructive apnea, requiring ventilation at home [40]. Due to the loss of consciousness, the EMS team was called to the child. The arriving team recognized asystole and began resuscitation. The rhythm has changed to PEA. After the child was transported to the hospital, the rhythm changed to ventricular tachycardia and VF with subsequent return of PEA. After 40 min of resuscitation, activities were discontinued, and she was pronounced dead; 2 min after the end of resuscitation, the child’s pulse returned spontaneously. The further fate of the patient remains unknown.

The literature on the problem also mentions the case of the Lazarus phenomenon in a 3-year-old boy suffering from cerebellar cancer. The patient in shock, with neutropenia, was admitted to the pediatric intensive care unit, where on the second day, bacterial abdominal inflammation and ascites developed, requiring laparotomy. Due to pleural effusion, chest drainage was performed, during which bradycardia and then cardiac arrest due to asystole occurred. Resuscitation lasting 25 min (previously intubated patient) and “aggressive” fluid therapy did not bring any result; 1 min after the end of rescue operations, a regular ECG rhythm was noticed on the monitor, and a pulse was detected. Unfortunately, 40 min later, refractory hypotension developed. Following a consultation with the family, life support was discontinued [6].

The literature also provides case reports of autoresuscitation in a group of patients less than a year old. One of them is a 9-month-old female infant [6]. Resuscitation activities were initiated by the girl’s father, who noticed apnea and imperceptible pulse. Resuscitation was carried out until the arrival of EMTs, who found PEA on arrival. During transport to the hospital, resuscitation was continued (and ventilation was performed at a rate of 38–52/min). Asystole was observed on arrival at the hospital. After 10 min, a FAST (Focused Assessment with Sonography for Trauma) ultrasound was performed to assess potential myocardial contractions that were not observed. The activities were stopped, but after 30 s, a tachyarrhythmia was noticed, and the presence of a pulse was confirmed. Repeated FAST examination showed the systolic function of the heart. The infant was taken to the pediatric intensive care unit, where a few days later, due to severe brain damage, life support was discontinued.

Another described example of the Lazarus phenomenon in an infant is an 11-month-old girl who was hospitalized due to chronic cardiac diseases: type 1 atrioventricular block, dilated cardiomyopathy, encephalopathy, and recurrent episodes of convulsions. Cardiac arrest with successful resuscitation occurred three times in the hospital. During the next fourth episode—in the asystole mechanism, CPR was initiated, but finally, the decision was made to discontinue it. The patient was disconnected from the monitor and extubated after 15 min. Gag reflexes were observed during the removal of the endotracheal tube. Respiration and pulse were checked successively, confirming their presence. Consciousness has also returned. After this episode, the girl was treated in a pediatric intensive care unit for a month. In the third month from the start of hospitalization, she was admitted to a chronic treatment center, where, 1.5 months later, in the course of dilated cardiomyopathy, her parents requested the DNR—Do Not Resuscitate [41]

## 8. Interesting Cases of Lazarus Syndrome

One of the special cases is a 97-year-old man for whom the patients will not to resuscitate was taken into account in the course of treatment—DNR. SCA took place, and, despite the lack of rescue measures, the patient spontaneously regained vital functions. He lived another 20 h [27].

Another interesting case took place in Japan, and it involved a 65-year-old hearing impaired who suffered from chronic gastritis. SCA occurred at the pre-hospital stage. Asystole was confirmed, and resuscitation was performed for 35 min. After the procedure, the patient’s remains were moved to the hospital mortuary. Later that day, a police officer arrived at the hospital mortuary to perform post-mortem procedures. He found this patient slightly moving and showing vital signs. He called the doctor, who confirmed agonal breathing and observed sinus rhythm in the ECG. The death of this patient was clearly confirmed 20 min earlier by the same doctor. The patient was immediately transported to the hospital ward but did not regain consciousness. On the fifth day, his condition deteriorated significantly, and he eventually died. The case of this patient in the cited literature is referred to as medical negligence caused by premature termination of resuscitation proceedings and subsequent transfer of the deceased for post-mortem examination within 14 h of death.

## 9. The Latest Cases of the Lazarus Phenomenon

The case described in 2019 by David J Sprenkeler [42] was an 86-year-old woman diagnosed with a third-degree atrioventricular block. After a sudden loss of consciousness, the patient was given transcutaneous stimulation lasting 1 h and 10 min, which was then discontinued due to poor prognosis. For 2 h from the end of stimulation, the agonal rhythm was <20/min, and complete heart block was also present. The decision was made to start palliative care, and 7 min later, the rhythm changed to asystole, which lasted for 4 min. Resuscitation procedures were not undertaken due to DNR. After 4 min, a nodal rhythm of 50/min appeared with a pulse, and after 2 h the patient regained consciousness. Two days later, the patient was implanted with a pacemaker and was discharged home in a fully functional neurological condition.

In 2020, three new cases of self-resuscitation were documented. One of them was cardiac arrest in the operating room during laparotomy in a 33-year-old patient who had previously had a ventriculoperitoneal shunt after meningioma surgery and now had an “acute abdomen” due to a ventriculoperitoneal shunt. Computed tomography of the head showed areas of acute infarction involving the brainstem and cerebellar hemispheres, as well as moderate enlargement of the ventricular system with left-sided intraventricular hemorrhage [43]. During the procedure, the patient developed bradycardia and asystole. Resuscitation was undertaken and continued for 30 min. As the asystole did not change, resuscitation activities were discontinued. The operating field was closed, and the patient was disconnected from the ventilator. Twenty minutes after ascertaining death, the operating team noted the return of breathing and spontaneous circulation (systolic blood pressure 60 mmHg, pulse 62/min, normal sinus rhythm, respiratory rate 12/min, oxygen saturation 88–90%). The team of surgeons decided to reopen the abdominal cavity. The patient was diagnosed with infarction of the distal ileum and right colon as a result of cicatricle fusion and an inflammatory response involving a ventriculoperitoneal shunt. After surgery, the patient was transferred to the intensive care unit, extubated on the 3rd day, and discharged from the ward one day later. Unfortunately, on the seventh day, the patient died.

The second case reported in 2020 was a 79-year-old patient who was injured in a car accident. After being transported to the hospital, she was intubated, but 5 min after intubation, PEA circulatory arrest occurred. Resuscitation was initiated, and the rhythm changed to VF. After 9 min of resuscitation, a decision was made to stop it, and 5 min later, echocardiography was performed, which showed no mechanical activity of the myocardium; 14 min after cessation of resuscitation, the patient’s breathing, pulse, and blood pressure returned (HR 132/min, RR 142/79 mmHg). The medical team undertook rescue activities to stabilize the patient’s condition; 10 h after the episode of autoresuscitation, cardiac arrest occurred again, but this time due to asystole. Resuscitation activities were not undertaken in agreement with the family [44].

In 2021, Martinez-Avilla M. et al. described the case of a 79-year-old woman treated at home for hypertension who was taken to a hospital with a Covid ward due to flu-like symptoms of high temperature, dehydration, general weakness, and respiratory problems. The SARS-CoV-2 infection was confirmed. Due to respiratory failure, the patient was intubated. Her general condition deteriorated, and she developed septic shock. On the 7th day of treatment, bradycardia developed, which turned into asystole. A 10-min CPR was performed. After this time, the circulation returned, but 15 min later, resuscitation was resumed, which was carried out for 40 min, and as a result of persistent asystole, further efforts were discontinued. Respiration and pulse returned 20 min after ascertaining death. The patient died after 14 days due to multiple organ failure, septic shock, and pneumonia in the course of COVID-19 [45].

A case of the Lazarus phenomenon described in 2022 was cardiac arrest in a 25-year-old woman [46] admitted to the hospital due to persistent vomiting and weight loss. The patient had undergone bariatric surgery 6 months earlier. On the 16th day of hospitalization, the patient’s condition deteriorated, and cardiac arrest occurred due to asystole. Resuscitation activities were initiated, lasting as long as 73 min. Due to the lack of effects, resuscitation was terminated, and the patient was pronounced dead. After 15 min, the family noticed eye movements, and circulation was restored. After 10 weeks of hospital stay, the patient with neurological deficit was discharged home.

In 2022, Kim HI. described a case of autoresuscitation, which he called the subtype of Lazarus Phenomenon, where electrocardiography in a patient appears spontaneously after cessation of death. This is about the transition from asystole to shockable rhythm. He described a 44-year-old female patient who developed ventricular tachycardia 6 min after death. Resuscitation efforts were unsuccessful, and the patient was pronounced dead. This case can be considered as incomplete Lazarus Syndrome as the pulse (or pulse and respiration) did not return; only a regular rhythm appeared on the ECG [47].

## 10. Summary

The article presents current data from the literature on the Lazarus phenomenon. The most common mechanisms which could lead to spontaneous recovery of vital functions were discussed. Additionally, according to the authors, some important and interesting cases of patients who “returned” to life were also presented.

In resuscitation proceedings, especially at the level of advanced activities, in order to avoid mistakes, great emphasis should be placed on the analysis and exclusion/treatment of the so-called reversible causes of cardiac arrest (ERC 2021). The analysis of the literature on the Lazarus phenomenon showed that the presence of a non-shockable rhythm [48,49,50] during CPR did not preclude autoresuscitation [50,51,52]. M. Grześkowiak showed the presence of 79% of non-shockable rhythms among all cases of the Lazarus phenomenon described so far. A systematic review of case reports of the Lazarus Phenomenon by M. Grześkowiak revealed a significant correlation between the duration of CPR and survival, with the Lazarus Phenomenon occurring more often among patients who were resuscitated for a longer period of time. Such a prolonged resuscitation lasting at least 30 min increases the patient’s chances of autoresuscitation. Based on the data from the literature, it can be assumed that the longer the cardiopulmonary resuscitation, the more often autoresuscitation can occur [25]. However, Gordon et al. did not provide a criterion for the duration of resuscitation and the subsequent occurrence of the Lazarus phenomenon [24]. The Lazarus phenomenon may also be caused by a delay in the action of drugs administered intravenously to peripheral vessels; therefore, in a patient with a central venous cannula, drugs should be administered via this route. The intraosseous route has a similar advantage over the supply of drugs to peripheral vessels.

All 76 reported cases of Lazarus Syndrome between 1982 and 2022 are summarized in Table 1 by gender, age, ECG rhythm, CPR time, time to autoresuscitation, and survival.

## 11. Conclusions

(1) Patients who have died after cessation of resuscitation should continue to be monitored (minimum ECG) for at least 10 min.
(2) During resuscitation, overventilation of the patient should be avoided due to the high risk of hyperinflation, which is mentioned as one of the causes of the Lazarus phenomenon.
(3) Continuing resuscitation without confirmation of myocardial contractility by echocardiography—which is recommended in the ERC 2021 guidelines as well as in the American Heart Association (AHA) 2020 guidelines—should not be abandoned too hastily.
(4) After prolonged CPR of at least 30 min, the patient’s chances of autoresuscitation are increased. It is especially common in the presence of non-shockable rhythms.
(5) The advanced age of the patient does not preclude the occurrence of the Lazarus phenomenon.
(6) To increase the awareness of the medical community, information about the phenomenon of Lazarus should be disseminated.
(7) We encourage you to report cases of the Lazarus phenomenon—this will allow you to create your own database and analyze the likely causes, which can be used to create guidelines to avoid premature pronouncement of death.
(8) At the current state of knowledge, the reasons for this phenomenon are not fully known, and continued follow-up and research are essential.
(9) The current scientific data presenting cases of Lazarus syndrome in given years do not constitute the final number of occurrences of autoresuscitation. Most likely, the presence of this phenomenon is more common. The extent of spontaneous recovery after death, along with the reporting of this phenomenon, still remains unknown.

## Figures and Tables

**Table 1:** Cases of Lazarus syndrome.

Year	Age of the Patient	Gender	Time of CPR (in Min)	Recognized Rhythm before Lazarus Syndrome	Time until Lazarus Syndrome Appears (in Min)	Survival Time	Reference
1982	80	M	20	A	5	35 days	[3,30]
	67	M	20	A	Unknown	15 days	
	68	F	Unknown	A	20	90 days	
	84	M	10	A	5	1 day	
1987	40	F	30	A	10	Unknown	[31]
1991	64	M	20	PEA	15	1 h	[8,32,36]
	36	F	25	PEA	3	6 months	
	49	M	30	A	5	15 days	
	Unknown	Unknown	Unknown	PEA	15	Complete recovery	
	75	F	20	A	5	Unknown	
	Unknown	Unknown	30	A	5	1 day	
1993	87	F	15	Unknown	Unknown	12 days	[4,53]
	75	M	23	A	5	Unknown	
1994	70	M	34	A	8	21 days	[33]
1996	55	M	30	A	7	3 days	[35,47]
	66	M	30	VF	5	Complete recovery	
1997	35	M	88	A	5	1 day	[48,49]
	54	F	50	PEA	1	93 days	
1998	67	F	43	A	5	9 days	[50,51]
	80	M	30	PEA	5	2 days	
1999	76	M	30	A	5	1 day	[52,54]
	59	F	15	PEA	2	30 min	
2001	66	M	18	A	10	13 days	[29,55,56]
	27	M	25	A	1	Unknown	
	93	F	6	Unknown	5	Unknown	
2002	Unknown	Unknown	15	PEA	Unknown	Unknown	[42,57]
	65	M	35	A	20	5 days	
2003	81	M	25	A	2	31 days	[58]
2004	81	F	13	A	Unknown	20 h	[59,60]
	94	F	40	PEA	3	21 days	
2005	41	M	15	A	23	5 days	[61,62,63]
	83	F	17	A	33	4 h	
	63	F	12	A	10	12 days	
2006	83	M	60	PEA	7	Unknown	[64,65]
	78	M	25	A	Unknown	19 h	
2007	52	F	CPR-NI	A	Unknown	Unknown	[9,66,67]
	47	M	26	A	15	3 months	
	85	M	CPR-NI	PEA	6	2 days	
2010	Unknown	Unknown	36	A	2	25 min	[68]
	84	M	15	PEA	5	Complete recovery	
2011	83	M	90	A	10	12 days	[6,28,40]
	3	M	25	A	1	40 min	
	9 months	F	Unknown	A	30 s	Unknown	
	10	F	40	PEA	2	Unknown	
2012	53	M	46	A	2	34 days	[69,70]
	65	M	55	A	40	13 days	
2013	89	F	18	A	5	370 min	[34,71]
	62	M	40	PEA	5	34 h	
2014	Unknown	Unknown	22	A	3	Full recovery	[34,72]
	21	M	30	A	10	57 days	
2015	67	M	47	PEA	5	1 day	[41,73]
	11 months	F	35	Unknown	15	4, 5 months	
2016	Unknown	M	CPR-NI	Unknown	1 min 42 s	108 s	[74,75,76,77]
	57	F	CPR-NI	Unknown	3	3 min	
	25	F	40	A	7 min	4 h	
	69	F	25	PEA	10	Unknown	
	44	M	80	PEA	5	3 days	
2017	69	M	40	A	180	10 days	[7,26,37]
	97	F	16	A	3	2 min	
	30	F	31	PEA	6	88 min	
	63	M	12	PEA	3	1 day	
	91	F	16	PEA	3	15 min	
	61	F	18	PEA	8	3 min	
	66	M	45	PEA	5	9 days	
2018	97	M	CPR-NI	A, VF	Unknown	20 h	[27,39]
	18 months	M	Unknown	PEA	6	Not full recovery (neurological dysfunction)	
2019	86	F	CPR-NI DNR procedure.	A	4	Unknown	[42]
2020	33	M	30	A	20	7 days	[43,44]
	79	F	9	PEA	14	10 h	
2021	79	F	10	A	20	14 days	[45,78,79,80]
	66	F	32	PEA	5	Unknown	
	16	M	CPR-NI DNR procedure.	A	Unknown	Unknown	
	2	M	CPR-NI	A	14	3 h and 35 min	
	18 month old	Unknown	20	Unknown	6	Unknown	
2022	25	F	73	A	15	Unknown	[46,81]
	44	F	Unknown	A	6	Unknown	

Abbreviations: M—Male, F—Female, A—Asystole, PEA—Pulseless Electrical Activity, VF—Ventricular Fibrillation, CPR—Cardio Pulmonary Resuscitation, DNR—Do Not Resuscitate, CPR-NI—CPR was Not Initiated.

## Data Availability

Data available in a publicly accessible repository. The data presented in this study are openly available in (repository name e.g., FigShare) at (doi), reference number (reference number).
